# The effects of age on skeletal muscle and the phosphocreatine energy system: can creatine supplementation help older adults

**DOI:** 10.1186/1476-5918-8-6

**Published:** 2009-12-24

**Authors:** Vincent J Dalbo, Michael D Roberts, Chris M Lockwood, Patrick S Tucker, Richard B Kreider, Chad M Kerksick

**Affiliations:** 1 Applied Biochemistry and Molecular Physiology Laboratory, Department of Health & Exercise Science, University of Oklahoma, Norman, Oklahoma, USA; 2 College of Education & Human Development, Texas A&M University, College Station, Texas, USA

## Abstract

Creatine supplementation has been found to significantly increase muscle strength and hypertrophy in young adults (≤ 35 yr) particularly when consumed in conjunction with a resistance training regime. Literature examining the efficacy of creatine supplementation in older adults (55-82 yr) suggests creatine to promote muscle strength and hypertrophy to a greater extent than resistance training alone. The following is a review of literature reporting on the effects of creatine supplementation on intramuscular high energy phosphates, skeletal muscle morphology and quality of life in older adults. Results suggest creatine supplementation to be a safe, inexpensive and effective nutritional intervention, particularly when consumed in conjunction with a resistance training regime, for slowing the rate of muscle wasting that is associated with aging. Physicians should strongly consider advising older adults to supplement with creatine and to begin a resistance training regime in an effort to enhance skeletal muscle strength and hypertrophy, resulting in enhanced quality of life.

## Introduction

Sarcopenia is an age-dependent loss of skeletal muscle mass resulting in reduced strength, limited mobility and increased injury risk[1]. Nearly 50% of older adults (≥ 60 yr) in the United States have been estimated to be sarcopenic, with approximately 20% classified as functionally disabled[2]. In 2000, direct health care costs of sarcopenia were estimated to be $18.5 billion. Interestingly, a 10% reduction in the prevalence of sarcopenia could save the United States $1.1 billion per year in health care costs[2]. The best treatments for reducing the effects of sarcopenia include resistance training[3,4] and testosterone administration[3]; however testosterone is often widely unavailable and may be associated with adverse effects in older adults[5]. Conversely, creatine supplementation may prove to be a safe and effective over-the-counter means to diminish age-related declines in muscle mass and strength as research has found creatine supplementation to increase strength[6] and type II muscle fiber diameter[7] independent of exercise. Moreover, creatine supplementation has repeatedly been found to increase performance in younger (≤ 35 yr) [8-15] adults, particularly when consumed in conjunction with a resistance training regimen. However, studies examining the effects of creatine supplementation in older adults (> 55 yr) have yielded apparently equivocal results [16-26]. This review will report on the effects of creatine supplementation on intramuscular high energy phosphates, skeletal muscle morphology and quality and quantity of life in older adults (> 55 yr). Furthermore, this review will attempt to elucidate why creatine supplementation may be effective in delaying sarcopenia, as well as discuss extraneous factors that may influence the efficacy of creatine supplementation in older adults.

### Aging and Skeletal Muscle Adaptations

At the cellular level sarcopenia is best described as a gradual loss of type II muscle fibers with a concurrent increase in intramuscular fat storage[27]. In 1983, Lexell et al. used autopsy analyses to demonstrate that aged men (70-73 yr) had approximately 110,000 less muscle fibers in the midsection of the vastus lateralis than younger men (19-37 yr), yielding a 23% decline in muscle fiber content. Furthermore, 80 yr old men possessed 30% less type II muscle fibers compared to younger counterparts[28]. Results from a different cross-sectional investigation[29] found that roughly 20-30% of lean body mass is lost between the third and eighth decade of life.

With age comes a shift in muscle fiber composition, resulting in a greater percentage of type I fibers which may affect energy metabolism[30]. Since type II fibers are predominantly involved in high force/power, short duration patterns of recruitment; potential alterations in energy-rich compounds could affect the phosphocreatine system and fast glycolysis. Moller et al.[30] were among the first to examine the effects of aging on energy rich compounds and the energy charge potential of the adenylate system. Specifically, Moller et al.[30] found intramuscular phosphocreatine levels to be 5% lower in older (52-79 yr) compared to younger (18-36 yr) adults, while intramuscular creatine levels were found to be 5% higher in older adults. However, the authors found ATP concentrations, the ratio of ATP/ADP and the energy charge potential to be unaffected with age. A later study in 61-80 yr old males found that cycling on a bicycle ergometer for 30 minutes, three times per week at workloads of 100-200 W to match each individuals maximal working capacity of 150-160 beats per minute, was enough to result in a significant decline in intramuscular creatine levels while significantly increasing the phosphocreatine/total creatine ratio[31]. Taken together these findings suggest exercise can increase intramuscular phosphocreatine levels in older adults, albeit no research has examined the effects of age on ATPase and creatine kinase which are likely responsible for the liberation of energy from ATP and the formation of phosphocreatine, respectively. If ATPase or creatine kinase is significantly reduced in aged populations the phosphocreatine energy system may be unable to perform optimally.

Further support for the ability of exercise to enhance the phosphocreatine energy system was demonstrated in humans by Moller et at[31] and in rats by Bastien et al. [32]. In each study intramuscular phosphocreatine was reduced with age in sedentary subjects. However, when a training program was initiated for a period of six[31] or twelve[32] weeks the phosphocreatine energy system was enhanced. Specifically, Moller et al.[31] found that six weeks of bicycle ergometer training in 61-80 yr old men resulted in a significant decrease in intramuscular creatine concentrations and significant increases in phosphocreatine/total creatine and ATP/ADP ratios.

### Why Creatine Supplementation Could Be Effective in Older Adults

It has been demonstrated that a reduction in cross sectional area (CSA) of type II fibers is primarily responsible for the decrease in muscle mass with aging and biopsy studies have found type IIA and type IIB muscle fiber CSA to decrease by 15-25% with age[33]. Creatine supplementation may aid in the retention of type II skeletal muscle fibers with age as research suggests creatine supplementation may be able to promote muscle hypertrophy through a variety of mechanisms including: cell swelling, which may act as a signal to reduce whole-body proteolysis and amino acid oxidation[34], alter the expression of myogenic transcription factors [15,35-37], increase satellite cell mitotic activity[38,39], and allow for increased exercise volume due to enhanced rates of ATP regeneration as a result of extending the duration of the phosphocreatine energy system[40,41]. Specifically, Sipila et al.[7] reported creatine supplementation increased type II skeletal muscle fiber diameter independent of a training stimulus. Furthermore, Willoughby and Rosene found that 6 g/day creatine supplementation, in conjunction with 12 weeks of resistance training, increased myosin heavy chain (MHC) isoform mRNA and protein expression to a greater extent than training alone in college-aged men[15], although this study design has not been sufficiently applied to older populations.

Currently the most popular mechanism to explain the efficacy of creatine supplementation is the enhancement of the phosphocreatine energy system, allowing users to maintain a greater work intensity for longer durations of time (i.e., increased total training volume). Research conducted by Moller et al.[30] found phosphocreatine and total adenine nucleotides to be significantly reduced in older compared to younger adults (creatine: 49.00 ± 0.85, 53.25 ± 1.98 mmol/kg dry weight; phosphocreatine: 75.50 ± 0.85, 72.73 ± 1.40 mmol/kg dry weight; total creatine: 124.40 ± 1.25, 126.28 ± 2.36 mmol/kg dry weight; data are reported as younger followed by older adults using means ± standard error); a finding supported by the work of Campbell et al.[42] (creatine: 53.52 ± 1.98, 66.3 ± 4.9 mmol/kg dry weight; phosphocreatine: 72.73 ± 1.40, 43.3 ± 2.8 mmol/kg dry weight; total creatine: 126.28 ± 2.36, 109.6 ± 4.8 mmol/kg dry weight; data are reported as younger followed by older adults using means ± standard error). However, the ATP/ADP ratio was not affected by age and the authors concluded ATP regeneration in older adults should be similar to that experienced in younger adults[30]. Since creatine supplementation has repeatedly been found to enhance intramuscular concentrations of phosphocreatine[43,44], enhance skeletal muscle strength[9,13,14,41,45] and hypertrophy[15,45] in college-aged individuals it is conceivable that older adults should experience comparable results.

Tables 1 and 2 were developed using a meta-analytical approach. Table 1 demonstrates the expected percent increase in resting high energy phosphate levels (total creatine, phosphocreatine, creatine and ATP) following creatine supplementation in younger and older adults. Table 2 identifies statistical differences in resting high energy phosphate concentrations between younger and older adults at rest prior to creatine supplementation. Furthermore, table 2 identifies statistical differences that are likely to exist between younger and older adults following a typical creatine supplementation regime. All referenced interventions supplemented subjects either through a loading phase (i.e., 20 g/d for 5 days) or through a 14-to-24 week supplementation protocol using 5 g/d and classified younger adults being between the ages of 18-36 yr and older adults being between the ages of 52-79 yr. Statistical significance was set at p < 0.05 and significance was determined utilizing separate independent samples t-tests.

**Table 1:** Typical intramuscular high energy phosphate levels in young and old

Variable	Young (baseline)^†^	% increase following supplementation^‡^	Old (baseline)^†^	% increase following supplementation^‡^
Total creatine	124.6 ± 2.7 (n = 96) [65-68]	21.7% (n = 36) [66-68]	129.8 ± 4.0 (n = 109) [20,25,30,31,42]	14.9% (n = 28) [20,25]
Phosphocreatine	78.2 ± 2.3 (n = 96) [65-68]	13.3% (n = 36) [66-68]	78.4 ± 4.1 (n = 109) [20,25,30,31,42]	8.2% (n = 28) [20,25]
Creatine	46.0 ± 2.3 (n = 96) [65-68]	39.3% (n = 36) [66-68]	51.4 ± 3.8 (n = 109) [20,25,30,31,42]	28.6% (n = 28) [20,25]
ATP	24.0 ± 0.6 (n = 96) [65-68]	4.2% (n = 36) [66-68]	20.0 ± 0.6 (n = 91) [20,25,30,31]	6.8% (n = 28) [20,25]

Values have been averaged from various investigations and are presented as: mean ± pooled S.E. values (n-size). References are presented as numbers next to value in brackets. Note that all studies assayed for the abovementioned metabolites (opposed to quantitation using ^31^P nuclear magnetic resonance spectroscopy) and these values are therefore expressed as mmol/kg muscle in dry weight.

^† ^Younger participants were between the ages of 18-36 years old, whereas older participants were between the ages of 52-79 years old.

^‡ ^All of the referenced interventions supplemented subjects either through a loading phase (i.e., 20 g/d for 5 days) or through a 14-to-24 week supplementation protocol using 5 g/d.

**Table 2:** Comparative analyses of high energy phosphate levels in younger and older adults prior to and following creatine supplementation

Variable	Young baseline^†‡^	Old baseline^†‡^	Significance	Theoretical results in young following supplementation	Theoretical results in old following supplementation	Potential statistical impact
TCr	124.6 ± 2.7	129.8 ± 4.0	< 0.001§	151.6 ± 2.7	149.1 ± 4.1	0.005 *
PCr	78.2 ± 2.3	78.4 ± 4.1	0.66	88.6 ± 2.3	84.8 ± 4.2	< 0.001 *
Cr	46.0 ± 2.3	51.4 ± 3.8	< 0.001§	64.1 ± 2.3	65.8 ± 3.9	0.03 *
ATP	24.0 ± 0.6	20.0 ± 0.6	< 0.001§	25.0 ± 0.61	21.4 ± 0.61	< 0.001 *

Baseline and theoretical values are presented as mean ± pooled S.E. values and were derived from Table 1.

TCr = Total creatine

PCr = Phosphocreatine

Cr = Creatine

ATP = Adenosine triphosphate

^† ^Younger participants were between the ages of 18-36 years old, whereas older participants were between the ages of 52-79 years old.

^‡ ^All of the referenced interventions supplemented subjects either through a loading phase (i.e., 20 g/d for 5 days) or through a 14-to-24 week supplementation protocol using 5 g/d.

§= Significant difference between young and old, p < 0.05.

* = Signifies a theoretical significant difference between young and old, p < 0.05.

In 2000, Tarnopolsky[46] hypothesized that creatine supplementation may provide more pronounced effects in older adults compared to the results typically experienced in younger adults. In support, numerous research studies have reported older adults to have lower intramuscular levels of total creatine (total creatine = creatine + phosphocreatine)[30,47] than younger counterparts. Individuals with lower endogenous total creatine levels seem to respond greater to creatine supplementation than individuals with higher endogenous total creatine levels[48,49], albeit these results have been equivocal[50].

#### Effects of Creatine Supplementation on the Phosphocreatine Energy System

In regards to active older adults, creatine supplementation may be able to have similar effects to that commonly displayed in younger adults supplementing with creatine. Smith et al.[48] used ^31^P nuclear magnetic resonance spectroscopy (MRS) to determine relative concentrations of intramuscular phosphocreatine in younger (30 ± 5 yr) and older (58 ± 4 yr; data is presented as means ± standard deviation) men while performing three sets of single-leg knee extensions, with 3 minute rest intervals between sets. Participants performed two sets of knee extensions for two minutes followed by one set of knee extensions until exhaustion. The exercise session was performed on three occasions: at baseline and following the five day consumption of 0.3 g•kg^-1^•day^-1 ^of a placebo (trial 1) and creatine (trial 2). Following the placebo, the authors found resting phosphocreatine concentrations (younger: 39.5 ± 5.1; older: 35.0 ± 5.2 mmol/kg) and the phosphocreatine resynthesis rate (younger: 23.2 ± 6.0; older: 18.1 ± 3.5 mmol•kg^-1^•min^-1^) to be significantly lower in older men compared to younger men. Following creatine supplementation resting phosphocreatine concentrations significantly increased in the younger (15%) and older men (30%). It is important to note that the phosphocreatine resynthesis rate also significantly increased in the older men to a level equivalent to the younger men (young: 24.3 ± 3.8; old: 24.2 ± 3.2 mmol•kg^-1^•min^-1^). This study demonstrates that creatine supplementation has a greater effect on phosphocreatine availability and resynthesis rate in older men than younger men.

In 2002, Rawson et al.[18] examined the effects of 5 days of creatine supplementation at a dose of 20 g/d on muscle phosphocreatine levels in younger (n = 8, 24 ± 1.4 yr) and older (n = 7, 70 ± 2.9 yr; data presented as means ± standard error) men. Baseline and post-supplementation measures of phosphocreatine were obtained using nuclear magnetic resonance spectroscopy. At baseline, phosphocreatine levels were significantly greater in the older (23.9 ± 0.7 mmol/kg wet weight) compared to the younger men (20.5 ± 0.7 mmol/kg wet weight). Following creatine supplementation, both groups experienced a significant increase in phosphocreatine (older: 7%, younger 35%). Since baseline differences were present between groups for intramuscular phosphocreatine levels, using baseline measures of phosphocreatine as the covariate, ANCOVA analyses revealed that younger men experienced a significantly greater increase in intramuscular phosphocreatine following supplementation compared to older men (younger: 27.6 ± 0.5; older: 25.7 ± 0.8 mmol/kg wet weight). Although creatine supplementation was not found to increase intramuscular phosphocreatine levels to the same extent in older men compared to younger men, creatine supplementation was responsible for physiologically significant increases in each population. Furthermore, it does not appear significant that the effect of creatine supplementation on intramuscular phosphocreatine levels in older adults was less dramatic than younger adults, as older adults had baseline phosphocreatine levels significantly closer to the maximal storage capacity of intramuscular phosphocreatine levels than younger adults. This study provides direct evidence that creatine supplementation can enhance intramuscular phosphocreatine levels in older adults, which may enhance the energy providing capacity of the phosphocreatine energy system in this population.

#### Creatine Supplementation and the Quality and Quantity of Life

Perhaps the most practical application of creatine supplementation in older adults is the potential to increase the quality and quantity of life[51,52]. Recently, two rodent models were used to examine the effects of long-term creatine supplementation on sarcopenia, overall health markers and survival. In 2005, Derave et al.[51] compared the muscle mass, morphology, contractility and metabolic properties of senescence-accelerated mice (SAMP8) who were creatine supplemented (2% of food intake) from weeks 10 to 60 of life (the equivalent of supplementation in humans from the ages of 6 yr to 30-40 yr of life[53]) against a control group. Creatine supplementation was found to have no significant effect on intramuscular ATP, total creatine, phosphocreatine, or absolute muscle mass of the soleus, extensor digitalis longus and tibialis anterior muscles. Further analyses revealed no significant differences between groups in absolute force or relative tension in the soleus or extensor digitalis longus. Finally, creatine supplementation had no significant effect on maintenance of muscle fiber size or distribution with age, leading the authors to conclude creatine supplementation is not an effective nutritional intervention to prevent sarcopenia in senescence-accelerated mice.

A more recent study examined the effects of long-term creatine supplementation in female wild-type C57BL/6J mice either fed a standard rodent diet or the same diet with 1% creatine, starting at the age of 52 weeks (the equivalent of starting creatine supplementation between the ages of 30-35 yr of life in a human[53]), 365 ± 2 days. The creatine-fed mice were found to have a significantly greater healthy life span (corresponding to a 9% increase), as defined by the age at which mice were classified as suffering from disease (creatine: 613 ± 84 days; control: 563 ± 95 days; results reported as mean ± standard error). Creatine supplementation was also found to enhance the overall lifespan (creatine: 716 ± 14 days; control: 692 ± 7 days) for the top 10% longest lived animals in each condition. Furthermore, creatine supplemented mice were found to have a trend increase in locomotor activity and mental health[52].

Results from Derave et al.[51] and Bender et al.[52] suggest that although long-term creatine supplementation may not significantly affect muscle morphology and intramuscular high-energy phosphate concentrations[51], creatine supplementation does appear to promote improved quality and quantity of life in mice, particularly when creatine supplementation began later in life and was continued until death[52]. Possible explanations to account for the results from Derave et al.[51] may be a down-regulation of creatine transporter protein concentration with prolonged exposure to supra-physiological exogenous creatine concentrations resulting from creatine supplementation. If correct, these results demonstrate the potential importance of cycling between periods of creatine use and disuse when consumed over long durations of time. Another explanation for the aforementioned results may involve the point of time within the lifespan in which the mice were supplemented. The pitfalls from Derave et al. may be due to the fact that creatine administration began early in life and was discontinued around the time of middle-age. Conversely, Bender et al. found creatine supplementation to enhance the quality and quantity of life in mice when creatine administration began at middle-age and was continued until death. Therefore, these results suggest that creatine supplementation may have a greater impact on overall health in mature adults rather than adolescence.

### Creatine Supplementation in Older Adults

Research appears to be equivocal regarding the effects of creatine supplementation in older adults. Creatine supplementation has been found to enhance muscle strength[24], endurance[24], power as measured by maximal isometric knee extension and flexion[22], lower body peak and mean power[22], lower body functional capacity using the sit-to-stand and tandem gait tests[22], body mass[22], fat-free mass[22,24], anaerobic power[26], work capacity[26] and has been shown to reduce muscle fatigue[16]. While other studies have found creatine supplementation to have no significant effect on body composition[16,23], maximal dynamic strength[16,23], dynamic or isometric endurance[23], time to fatigue[54] and isometric strength[21]. However, variable creatine supplementation protocols and different methodologies may account for the equivocal findings between studies. See Additional Files 1 and 2) for a summary of effective and ineffective studies conducted on creatine supplementation in older adults.

#### Low Dose Creatine Supplementation and Functional Tasks

A placebo-controlled study conducted by Gotshalk et al.[22] found creatine supplementation at a dose of 0.3 g•kg^-1^•d^-1 ^to significantly increase body mass, 1 repetition maximum (RM) leg and bench press strength, isometric knee extension and flexion, lower body average peak and mean power, and improve performance in the sit-to-stand and tandem gait tests in older men (57-72 yr). In 2008, Gotshalk et al.[17] examined the effects of creatine supplementation in 30 older women (58-71 yr). Participants were assessed on the sit-to-stand test, 1 RM bench press and leg press, isometric hand-grip test, tandem gait test, and upper and lower body ergometer tests prior to and following supplementation with 0.3 g•kg^-1^•d^-1 ^of a placebo for seven days followed by supplementation at a dose of 0.3 g•kg^-1^•d^-1 ^of creatine for seven days. Compared to the placebo condition, consuming creatine resulted in a significant increase in body mass and fat-free mass, along with a significant increase in 1 RM bench press and leg press, and increased performance on the tandem gait test. The investigations by Gotshalk et al.[17,22] are important for several reasons. First, creatine supplementation was found to improve performance in several practical measures of quality of life including strength and functional capacity tests (sit-stand and tandem gait) in older populations of men and women. Second, participants exposed to a low dose creatine regimen (0.3 g•kg^-1^•d^-1^) increased body mass and performance, suggesting low dose creatine supplementation can be effective in older populations. Finally, creatine supplementation improved performance independent of training and since the experimenters had participants perform six familiarization protocols it is not likely that any performance improvements were due to a learning effect.

Another study[24] compared the effects of resistance training and 10 weeks of supplementation of either creatine or a placebo administered at a dose of 0.3 g•kg^-1^•d^-1 ^for the first five days, then 0.07 g•kg^-1^•d^-1 ^for 65 days in older adults (71.0 ± 1.5 yr). Training consisted of 36 resistance training sessions in which participants performed three sets of 10 repetitions for the bench press, bicep curl, back extension, hip (extension, flexion, adduction and abduction), leg flexion, knee extension and leg press. When compared to the placebo group, creatine supplementation significantly increased lean body mass, 1 RM leg press and knee extension performance, leg press and knee extension endurance, and average lower body power as assessed by three sets of 10 repetitions of isokinetic knee extension/flexion at 60°•s^-1 ^with one-minute rest periods between sets. There were no differences between groups for fat mass, percent body fat, 1 RM bench press or bench press endurance. Of particular interest was the finding that lower body muscle strength and endurance was significantly enhanced with creatine supplementation while having no significant effect on maximal upper body strength or endurance. These findings suggest that in older adults, creatine supplementation may be most effective at enhancing performance of large muscle groups and may explain the non-significant increases in strength using the elbow flexors in other investigations[16,21].

In contrast, Berman et al.[23] examined the effects of consuming an oral creatine supplement or placebo at a dose of 20 g/day for five days and 3 g/d for an additional 47 days in 32 older (67-80 yr) men and women. Participants were randomly divided into one of four groups: creatine-no training, placebo-no training, creatine-exercise or placebo-exercise. Participants in the training groups performed three sets of eight repetitions for leg press, leg extension and chest press, three days per week for seven weeks. Anthropometric measures included assessment of percent body fat via skin-fold assessment and an estimation of lower limb muscle CSA by correcting for thickness of skin and subcutaneous fat by skin-fold measurements. Following seven weeks of supplementation and training, creatine ingestion was found to have no effect on body mass, body fat, or lower limb muscle volume. Furthermore, creatine was found to have no effect on muscle strength and muscle endurance as supplementation did not significantly improve 1 or 12 RM performance for the leg press, leg extension or seated chest press. However, a major limitation of this study was allowing all training groups to self-select the progression of their resistance training program, rather than following a standardized rate of progression during the course of the study. One of the primary mechanisms by which creatine supplementation has been proposed to improve performance is through enhancement of the phosphocreatine energy system[41] allowing users to train at higher volumes. It is unlikely older men and women were self-selecting loads that would optimize the effects of creatine supplementation for muscle strength and endurance enhancement. Despite the short-comings of the study design, participants in the creatine-exercise group performed better, albeit it not significantly different than participants in the placebo-exercise group on each of the muscle strength and muscle endurance measures.

In 1999, Rawson and Wehnert[16] examined the effects of consuming creatine or a placebo at a dose of 20 g/day for 10 days followed by 4 g/d for 20 days on body composition and strength independent of training. Creatine supplementation was found to have no effect on body mass, body density, fat-free mass or elbow flexor strength. However, creatine was effective at enhancing lower body muscle endurance as determined by assessing the percent change in total peak torque generated during leg extensions on an isokinetic dynamometer. Participants performed five sets of 30 maximal voluntary contractions at 180°•s^-1^. The finding that creatine supplementation in the absence of training does not improve strength is not surprising. However, it is plausible that a significant effect may have been observed if strength testing involved exercises requiring large muscle groups, such as using a chest or leg press, particularly since lower body muscle endurance was found to be enhanced.

#### Creatine Supplementation and Skeletal Muscle Fatigue

Wiroth et al.[26] examined the effects of 15 g/day of creatine or a placebo on maximal pedaling performance in older men. Participants were separated into three groups: older sedentary (n = 14, 70.1 ± 1.2 yr; data represented as means ± SE), older trained cyclists (n = 14, 66.4 ± 1.4 yr) and younger sedentary (n = 14, 26.0 ± 1.2 yr). Prior to and following supplementation all participants performed five maximal exertion 10-second sprints on a cycle ergometer, with 60-second rest intervals of passive recovery between sets. Creatine supplementation was found to significantly enhance maximal power and total work in the younger and older sedentary groups. Results of this study suggest short-term creatine supplementation can enhance anaerobic power and work capacity in younger and older adults and that short-term creatine supplementation is able to significantly improve performance in untrained participants. However, creatine supplementation may have a less pronounced affect on trained individuals or trained individuals may require longer supplementation periods for creatine to significantly improve performance.

In a double-blind, randomized, cross-over design Stout et al.[55] examined the effects of consuming creatine or a placebo at a dose of 20 g/d for seven days followed by a dose of 10 g/d for an additional seven days in older men and women (74.5 ± 6.4 yr; data reported as mean ± standard deviation). The experimenters employed a 4-6 week washout period prior to cross-over to the other condition. Also, testing was conducted prior to and following each supplementation period. There were no differences between groups for body mass or performance on the sit-to-stand test; however, participants performed significantly better on grip strength and physical working capacity fatigue threshold tests when consuming creatine compared to the placebo. Results from this investigation provide further evidence that short-term creatine supplementation may enhance strength and delay neuromuscular fatigue which are important functions for maintaining health and independent living with age.

Jakobi et al.[54] examined the effects of creatine supplementation on neuromuscular properties and fatigue in 12 older (65-82 yr) men. The five-day supplementation protocol consisted of two groups of men receiving either 20 g/day of both creatine and maltodextrin (n = 7; 4 × 5 g creatine + 5 g maltodextrin) or a maltodextrin placebo (n = 5; 4 × 5 g maltodextrin). Maximal isometric voluntary force, muscle activation and surface electromyography were measured in elbow flexor muscles at baseline, during a fatiguing task, and over a 10 minute recovery period. Compared to placebo, creatine supplementation was found to have no effect on body mass, maximal isometric voluntary force or muscle activation. Creatine supplementation was also found to have no effect on time to fatigue, decline in maximal isometric voluntary force, muscle activation or contractile properties during the fatiguing protocol. Similarly, creatine provided no beneficial effect on rates of recovery, voluntary force or stimulated contractile force during recovery. Results from this investigation, albeit limited in sample size and statistical power, suggests that short-term (5 days) creatine supplementation does not influence isometric performance of the elbow flexors in older men.

In 2000, Rawson and Clarkson[21] examined the effects of creatine and sucrose (20 g/d of creatine and 4 g/d of sucrose) or a placebo (24 g of sucrose) on isometric strength, isokinetic skeletal muscle endurance and body mass in 17 older (60-78 yr) men. Results indicate that creatine supplementation significantly increased body mass while having no significant effect on isometric strength as determined by elbow flexion. There was a significant interaction effect, as creatine supplementation significantly enhanced muscle endurance as determined from an intermittent fatigue test of the knee extensors in which participants performed three sets of 30 repetitions, with 60-second rest periods between sets. However, the authors concluded that the significant effect on knee extensor performance was not meaningful as the creatine group experienced a small non-significant increase and the placebo group experienced a small non-significant decrease in performance. Results from this investigation suggest a loading phase of 20 g/d of creatine and 4 g/d of sucrose is able to significantly increase body mass and possibly muscle endurance.

#### Effects of Creatine Supplementation on Skeletal Muscle Morphology

Brose et al.[25] examined the effects of 14 weeks of resistance training on male and female participants supplemented with either 5 g/day creatine plus 2 g/day of dextrose or 7 g/day of a dextrose only placebo. The resistance training regime consisted of three exercise sessions per week in which participants performed a circuit of 12 exercises prescribed to train the major muscle groups of the upper and lower body. Training progressed from one set of each exercise at 50% of initial 1 RM, to three sets at 80% 1 RM. The 1 RM was re-evaluated every two weeks and the training loads were adjusted to meet the new load demands. Moreover, muscle biopsies were obtained prior to and following supplementation for assessment of mean fiber area and percentage of fiber distribution of type I, IIA and IIX skeletal muscle fibers. Additional muscle analyses included assessment of creatine, phosphocreatine, total creatine and ATP concentrations. Training resulted in significant increases in all measures of strength, functional tasks, and muscle fiber area in both the creatine and placebo groups. Compared to the placebo, creatine supplementation was found to significantly increase intramuscular total creatine, total body mass and fat-free mass while increasing isometric knee extension strength in men and women, and dorsiflexion strength in men. Results from this study suggest an adequate resistance exercise program alone can reverse the effects of sarcopenia; however, consuming a low dose of creatine during training may enhance the effects of training. Specifically, training plus creatine may increase both muscle mass and strength in older men and women to a greater extent than resistance training alone.

In 2003, Eijnde et al.[20] examined the effects of six months of training plus 5 g/day creatine supplementation or placebo in 46 older (55-75 yr) men. Pertinent variables assessed included body weight, fat-free mass, percent body fat, maximal isometric strength, and intramuscular concentrations of ATP, creatine, phosphocreatine and total creatine. Creatine supplementation was found to have no significant effect on body mass, percent body fat or fat-free mass compared to the placebo condition. Creatine supplementation did not affect intramuscular stores of ATP, creatine, phosphocreatine and total creatine when compared to the placebo. Flaws in study design include a resistance training protocol designed to stimulate improved muscular endurance (two sets of 30 repetitions of seven exercises), but baseline and post-test measures assessed maximal strength. Moreover, the fact that creatine supplementation did not improve intramuscular levels of high-energy phosphates to a greater extent than the placebo may suggest there were a large number of non-responders to creatine supplementation in the investigation. The possibility also exists that the training necessary to maximize the effects of creatine supplementation may need to be of a higher intensity than that designed to promote skeletal muscle endurance.

#### Combined Effects of Creatine and Protein Supplementation

Most recently, Candow et al.[56] conducted a study utilizing 35 older (59-77 yr) men who were resistance trained three days per week for 10 weeks. Participants were randomly assigned in a double-blind fashion to one of three groups: creatine-protein (0.1 g•kg^-1 ^of creatine + 0.3 g•kg^-1 ^of protein), creatine (0.1 g•kg^-1 ^of creatine) or a placebo. Supplements were only consumed on training days. Creatine and creatine-protein supplementation resulted in significantly greater gains in body mass, total muscle thickness and less bone resorption assessed by cross-linked N-telopeptides of type I collagen (NTx) when compared to the placebo group. Further analyses revealed the creatine-protein group increased bench press strength to a significantly greater degree than the creatine and placebo groups. Results from this investigation demonstrate that low-dose creatine consumed only three days per week can help improve health in older populations when used in conjunction with a resistance training program. Furthermore, the benefits of creatine may be enhanced in older adults when consumed with protein.

#### Rates of Muscle Loss Following Cessation of Creatine Supplementation

Results from numerous studies have found creatine supplementation to be effective at enhancing muscle function in older adults[17,22,24-26,55] but the only study to examine the effects of ceasing creatine supplementation while maintaining resistance training in older adults was conducted by Candow et al.[19]. Participants supplemented with 0.3 g•kg^-1^•d^-1 ^for a period of five days and 0.07 g•kg^-1^•d^-1 ^for 11 weeks with either creatine or a placebo. Participants in the creatine group experienced significantly greater gains in body mass, total muscle thickness and less bone resorption than participants consuming the placebo. A sub-group of participants supplementing with creatine or the placebo then trained for an additional 12 weeks at a reduced training volume (33% lower). Cessation of creatine supplementation was found to have no effect on strength or lean tissue mass. Muscle endurance was significantly decreased following the cessation of creatine, but the rate of loss was similar between participants in the creatine and placebo groups. Since participants supplementing with creatine experienced a greater increase muscle thickness than participants supplementing with a placebo while resistance training at a volume/intensity designed to stimulate skeletal muscle hypertrophy and upon the cessation of creatine supplementation and beginning a training regime at a lower intensity there were no differences between groups for loss of muscle function or size these results suggest that older adults who supplement with creatine should experience greater improvements in strength and hypertrophy over time than older adults who only resistance train.

#### Implications

Collectively, creatine supplementation studies in older men and women[17,21,22,24-26,55] suggest that doses as low as 0.3 g•kg^-1^•d^-1^, for a period of as little as seven days[17,22], can significantly enhance muscle strength and functional capacity, independent of training. Furthermore, numerous studies have found that short-term creatine supplementation ranging from five days[21] to fourteen weeks[25] can result in significant increases in body mass[17,21,22,25], fat-free mass[17,22,24,25] and strength[17,21,22,24,25,55]. For example, Brose et al.[25] reported that 5 g/day of creatine plus 2 g/day dextrose, for 14 weeks, significantly increased intramuscular total creatine levels. As a result, creatine supplementation appears to be an effective intervention for maintaining strength, body mass, lean mass and functional capacity with age.

### Potential Problems with Creatine Supplementation in Older Adults

The use of questionable methodologies may account for the lack of significant performance improvements following creatine supplementation in older adults, but physiological variables may also account for the non-significant findings. For example there are three proposed inter-individual responses to creatine supplementation: 1) responders, individuals who experience a > 20 mmol·kg^-1 ^dw increase in total creatine[57,58], 2) quasi responders, individuals who experience a 10-20 mmol·kg^-1 ^dw increase in total creatine[58] and 3) non-responders, individuals who experience < 10 mmol·kg^-1 ^dw increase in total creatine[57].

Syrotuik and Bell[58] conducted a study to determine the physiological profile of responders and non-responders to creatine supplementation. Participants consisted of younger males (22.7 yr) who supplemented with creatine for 5 days at a dosage of 0.3 g•kg^-1^•d^-1^. Of the 11 participants 3 were responders, 5 were quasi responders and 3 were non-responders. There was a descending trend regarding muscle fiber composition, body mass and fat free mass between the three groups. Responders had the highest percentage of type II fibers (63.1%), followed by quasi responders (45.5%) and non-responders (39.5%). Responders and quasi responders had a greater muscle cross-sectional area than non-responders pre and post creatine supplementation. Responders, quasi responders and non-responders had mean cross-sectional areas for type I (1,509; 1,270; 900 μm^2^), type IIA (1,807; 2,238; 1,377 μm^2^) and type IIB (1,695; 1,740; 1,213 μm^2^) fibers, respectively. Responders had greater mean muscle fiber increases than non-responders, type I (320 verse 60 μm^2^), type IIA (971 verse 46 μm^2^), and type IIB (840 verse 78 μm^2^). The authors concluded the primary determining factors influencing the effectiveness of creatine supplementation were muscle fiber distribution and muscle CSA[58]. In terms of effects on body composition and strength responders had the greatest increase in total body mass (2.4 kg) and fat free mass (2.0 kg), followed by quasi responders (2.1 kg and 1.9 kg) and non-responders (1.9 kg and 1.7 kg). Responders improved maximal leg press performance by 25.8 kg followed by quasi responders (2.5 kg) and non-responders (2.0 kg).

Results from the Syrotuik and Bell[58] investigation do not bode well for the practicality of creatine supplementation in older adults, a population known to have a greater percentage of type I muscle fibers[59,60] and reduced muscle CSA[33,61,62]. As a result older adults may be less likely to respond to creatine supplementation compared to younger adults. Additionally, creatine uptake is enhanced when consumed in conjunction with carbohydrates. This is problematic as aging is associated with glucose intolerance and a decline in insulin-stimulated transport[63]. Moreover, the saturable component of creatine transport is related to cell age and is greater in young cells[64]. Taken together older adults may not be as responsive to the traditional creatine dosing regimen typically employed in younger adults (loading phase: 20 g/d, maintenance phase: 5 g/d) and may require longer supplementation periods or may need to consume higher doses of creatine to reach saturation levels in muscles[18]. However, this hypothesis has not been supported by the majority of research conducted in older adults as dosing interventions as low as 0.1 g•kg^-1 ^three days per week for 10 weeks combined with resistance exercise have been found to significantly increase body mass and total muscle thickness[56]. While a dose of 5 g/d of creatine with 2 g/d of dextrose for 14 weeks has been found to increase intramuscular total creatine concentrations in older (> 65 yr) men and women[25].

Finally, the theoretical increase in total creatine following a standard dosing protocol of creatine is 19.3 mmol/kg in older and 27.0 mmol/kg in younger adults (Table 2). These differences suggest a greater responder rate in younger compared to older adults and/or an increased non-responder rate in older adults. This theory is supported by Greenhaff et al.[57] who suggest it may be necessary to increase intramuscular total creatine by approximately 20 mmol·kg^-1 ^dry weight (dw) to obtain significant performance improvements from creatine supplementation. Following this theory Greenhaff et al.[57] suggest that 20%-30% of younger adults fall into the category of non-responders following five days of creatine supplementation at a dose of 20 grams per week. Thus, it is possible that the greater non-responder rate in older adults may account for the lack of significant performance improvements observed in some studies; particularly those with small sample sizes and statistical power[58].

## Conclusions

Despite physiological adaptations that occur with aging that may reduce the effectiveness of creatine supplementation, well designed studies have found creatine supplementation to safely enhance muscle strength[17,22,24-26,56], hypertrophy[17,22,24-26,56], endurance[22,24,55] and performance in functional tasks[17,22] in older adults. Of equal interest, such results have been observed in response to relatively short interventions (5-7 days of creatine supplementation). Therefore, creatine supplementation should be strongly considered as a safe, inexpensive and effective nutritional intervention to help slow the rate of muscle wasting with age, particularly when consumed in conjunction with a resistance training regimen. Future research should examine factors that can affect creatine transport into muscle, such as creatine transporter protein concentrations and uptake from oral ingestion. Researchers also need to examine the effects of age on ATPase and creatine kinase levels, which are integral enzymes needed for phosphocreatine energy system functionality. Finally, research in older populations should focus on the ideal dosing and timing of creatine supplementation, along with the practicality of combining creatine consumption with protein, beta-alanine and/or beta-hydroxy beta-methylbutyric acid (HMB) as each may benefit older adults.

## Competing interests

The authors declare that they have no competing interests.

## Authors' contributions

VJD developed the concept and wrote the review paper. MDR contributed in writing the review and aided in the development of the tables. CML, PST, RBK and CMK critically reviewed the manuscript. All authors read and approved the final manuscript.

## Supplementary Material

Additional file 1 **Table 3. **Creatine supplementation enhances performance in older adults Click here for file

Additional file 2 **Table 4** Creatine supplementation does not enhance performance in older adults Click here for file
