# Supplementary material for: The effects of age on skeletal muscle and the phosphocreatine energy system: can creatine supplementation help older adults
Source: Dyn Med. 2009 Dec 24;8:6. doi: 10.1186/1476-5918-8-6 (PMC2807421; doi:10.1186/1476-5918-8-6)
Supplement: Additional file 1 — Table 3. Creatine supplementation enhances performance in older adults [file 1476-5918-8-6-S1.PDF]

Table 3. Creatine supplementation enhances performance in older adults

Author	Training and Familiarization Protocol	Study Design and Supplement Protocol	Significant Results
Wiroth et al., 2001 [26]	No training.<br><br>Testing conducted at baseline and following supplementation. For testing participants performed five all out 10-s sprints followed by 60-s of passive recovery on a cycle ergometer.	*Double-blind, randomized design in each group of men.* Three groups: older sedentary, older trained and younger sedentary. Participants in each group consumed either 3 x 5 $g \bullet day^{-1}$ of Cr or 3 x 10 $g \bullet day^{-1}$ of a PLA.	↑ maximal power and work done in the sedentary older and sedentary younger groups
Gotshalk et al., 2002 [22]	No training.<br><br>There were six familiarization sessions followed by 3 testing sessions: baseline, testing following 7 days of no supplementation, and testing following 7 days of supplementation.	*Double-blind, placebo controlled design with repeated measures*. Supplemented 18 males with 0.3 $g \bullet kg^{-1} \bullet d^{-1}$ of Cr or PLA for 7 days.	↑ in 1 RM leg press and bench press with Cr<br><br>↑ isometric knee extension and flexion with Cr<br><br>↑ lower body average peak power and mean power with Cr<br><br>↑ performance in sit-to-stand and tandem gait test with Cr<br><br>↑ body mass and fat-free mass with Cr

Stout et al., 2007 [55]	No training.<br><br>Baseline testing was conducted along with post-testing following each supplementation period.	*Double-blind, cross-over, randomized design.* A total of 15 men and women were assigned to consume Cr or a PLA at a dose 20 g/d for seven days followed by a dose of 10 g/d for seven days. Following a 4-6 week washout participants crossed-over to the other condition.	↑ grip strength with Cr<br><br>↑ physical working capacity fatigue threshold with Cr<br><br>↔ sit to stand test with Cr<br><br>↔ body weight with Cr
Gotshalk et al., 2008 [17]	No training.<br><br>There were six familiarization sessions followed by 3 testing sessions: baseline, testing following 7 days of no supplementation, and testing following 7 days of supplementation.	*Double-blind, placebo controlled design with repeated measures*. Supplemented 30 women with 0.3 $g•kg^{-1}•d^{-1}$ of Cr or PLA for 7 days.	↑ 1RM bench press and leg press with Cr<br><br>↑ performance on functional tandem gait test<br><br>↑ body mass and fat-free mass with Cr
Chrusch et al., 2001 [24]	One familiarization session followed by 36 training sessions consisting of 12 exercises, each performed for 3 sets of 10 reps, 3 days per week.<br><br>Testing occurred at baseline, 28 days after supplementation and during the last week of training.	*Double-blind administration with 30 older men.* Supplemented with Cr or PLA at a dose 0.3 $g•kg^{-1}•d^{-1}$ for the first 5 days, then 0.07 $g•kg^{-1}•d^{-1}$ for 65 days.	↑ in 1 RM leg press and knee extension with Cr<br><br>↑ in leg press and knee extension endurance with Cr<br><br>↑ in average power with Cr<br><br>↑ in lean body mass with Cr<br><br>↔ between groups for changes in fat mass, % body fat, bench press 1RM and bench press endurance

Brose et al., 2003 [25]	Participated in a whole body resistance training program 3 days per week for 14 weeks. Testing was conducted at baseline and following 14 weeks of training.	*Double-blind, randomized design a total of 28 men and women.* Supplemented with Cr: 5 g/d with 2 g/d of dextrose or PLA: 7 g of dextrose.	↑ isometric knee extension with Cr<br><br>↑ doriflexion strength in men with Cr<br><br>↑ intramuscular total creatine with Cr<br><br>↑ total body and fat-free mass with Cr
Candow et al., 2008 [56]	Participants resistance trained 3 days per week for 10 weeks.	*Double-blind, placebo controlled, randomized design*. A total of 35 men were assigned to a Cr-PRO (0.1 $g \cdot kg^{-1}$ Cr + 0.3 $g \cdot kg^{-1}$ PRO), Cr, or PLA group. Supplements were only consumed on training days.	↑ Body mass, total muscle thickness and less bone resorption with Cr and Cr-PRO<br><br>↑ bench press strength with Cr-PRO

↑,↓,↔ Signifies increase, decrease, or no change compared to a control/placebo condition
Cr: creatine, PLA: placebo, RM: repetition maximum, PRO: protein
