# Supplementary material for: The effects of age on skeletal muscle and the phosphocreatine energy system: can creatine supplementation help older adults
Source: Dyn Med. 2009 Dec 24;8:6. doi: 10.1186/1476-5918-8-6 (PMC2807421; doi:10.1186/1476-5918-8-6)
Supplement: Additional file 2 — Table 4 Creatine supplementation does not enhance performance in older adults [file 1476-5918-8-6-S2.PDF]

Table 4. Creatine supplementation does not enhance performance in older adults

Author	Training and Familiarization Protocol	Study Design and Supplement Protocol	Significant Results
Rawson et al., 1999 [16]	No training.<br><br>Testing occurred at baseline and following 10 and 30 days of supplementation.	*Double-blind, placebo controlled, randomized design.* Supplemented 20 males with Cr or PLA at a dose of 20 g/day for 10 days followed by 4 g/d for 20 days.	↔ strength of elbow flexors with Cr<br><br>↔ body mass, body density or fat-free mass with Cr<br><br>↑ performance in leg fatigue task with Cr
Rawson et al., 2000 [21]	No training.	*Double-blind, placebo controlled, randomized design.* Supplemented 17 males with 20 g of Cr and 4 g of sucrose (Cr) or 24 g of sucrose (PLA) for 5 days.	↔ isometric strength of elbow flexors with Cr<br><br>↑ isokinetic knee extension with Cr, but authors concluded not meaningful<br><br>↑ body mass with Cr
Jakobi et al, 2001 [54]	No training.<br><br>Testing consisted of a familiarization protocol followed by baseline testing and post-testing.	*Placebo controlled, randomized design*. Supplemented 12 men with either Cr and maltodextrin (20 g/d of each) or a PLA (20 g/d of maltodextrin).	↔ MVC or muscle activation with Cr<br><br>↔ time to fatigue, decline in MVC, muscle activation or contractile properties with Cr<br><br>↔ rate of recovery, voluntary force or stimulated contractile force during recovery with Cr<br><br>↔ body mass with Cr

Berman et al., 1998 [23]	Following four familiarization sessions a whole body resistance training program was initiated that involved lifting 3 days per week for 7 weeks. Testing was conducted at baseline and following 7 weeks of training.	*Double-blind, placebo controlled, randomized design with 32 men and women.* Four groups: Cr-control, PLA-control, Cr-exercise, PLA-exercise. Supplemented with Cr or PLA at 20 g/day for 5 days and 3 g/d for 47 days.	↔ body mass, body fat or lower limb muscle volume with Cr<br><br>↔ 1 and 12 RM and isometric intermittent endurance tests for bench press, leg press and leg extension with Cr.
Eijnde et al., 2003 [20]	Training consisted of 24 minutes of endurance training and a whole body resistance training program. Testing was conducted at baseline, 6 months following training and 1 year post-training in a subgroup of 20 participants.	*Double-blind, placebo controlled, randomized design.* Supplemented 46 men with either Cr of PLA at a dose of 5 g/d for 6 months.	↔ maximal isometric strength with Cr<br><br>↔ Body mass with Cr<br><br>↑ total creatine in Cr group after 6 months

↑,↓,↔ Signifies increase, decrease, or no change compared to a control/placebo condition
Cr: creatine, PLA: placebo, RM: repetition maximum, MVC: maximal isometric voluntary force
